# The impact of physical exercise interventions on social, behavioral, and motor skills in children with autism: a systematic review and meta-analysis of randomized controlled trials

**DOI:** 10.3389/fped.2025.1475019

**Published:** 2025-02-27

**Authors:** YanAn Wang, Guoping Qian, Sujie Mao, Shikun Zhang

**Affiliations:** ^1^ Student Affairs Office, Jiangsu Police University, Jiangsu Police Institute, Nanjing, Jiangsu, China; ^2^ Department of Fitness, Gdansk University of Physical Education and Sport, Gdansk, Poland; ^3^ Graduate School, Harbin Institute of Physical Education, Harbin Sport University, Harbin, Heilongjiang, China

**Keywords:** autism, motor skills, children, social skills, exercise intervention, physical activity

## Abstract

**Background:**

Autism Spectrum Disorder (ASD) is a neurodevelopmental disorder characterized by social impairments, sensory processing issues, repetitive behavior patterns, motor abnormalities, and executive function impairments.

**Objective:**

To systematically review and meta-analyze the effects of various exercise modalities on flexibility and cognitive control, social skills, behavioral problems, motor skills, and coordination in children with ASD, providing scientific evidence for clinical practice to guide effective exercise interventions for children with ASD.

**Methods:**

Literature searches were conducted in PubMed, EMbase, Cochrane Library, EBSCOhost, and Web of Science databases, covering the period from database inception to February 15, 2024. Inclusion criteria included studies involving children with ASD, any form of exercise intervention, reporting at least one ASD-related outcome, and designed as randomized controlled trials (RCTs) or quasi-experimental studies. Exclusion criteria included reviews, conference abstracts, commentary articles, and studies lacking sufficient statistical data for meta-analysis. Study quality was assessed using the PEDro scale. Effect sizes were calculated using standardized mean differences (SMD). Heterogeneity was assessed with the *I*^2^ statistic. Multiple subgroup analyses were conducted, and publication bias was evaluated using Begg's Test and Egger's Test.

**Results:**

23 RCTs were included in this study, showing positive effects of exercise interventions in various domains.Upper grade students showed significant improvement in flexibility and cognitive control (SMD = −0.282, *p* = 0.161). Lower grade children showed the most significant improvement in motor skills and coordination (SMD = 0.475, *p* = 0.043). Preschool children showed significant enhancement in social skills (SMD = 0.312, *p* = 0.041). Behavioral problems improved significantly across all age groups (SMD = −0.674, *p <* 0.001). Martial arts and ball games were particularly effective in enhancing these domains, and appropriate periodic exercise interventions effectively improved various abilities in children with ASD. Results varied across different ages and intervention types.

**Conclusion:**

Exercise interventions significantly improve flexibility, cognitive control, motor skills, coordination, social skills, and behavioral problems in children with ASD. This study supports exercise interventions as an effective method to enhance multiple abilities in children with ASD and emphasizes the importance of designing personalized intervention programs tailored to different ages and needs. Future research should focus on larger sample sizes and long-term follow-ups to confirm the sustainability and generalizability of intervention effects.

## Introduction

Autism Spectrum Disorder (ASD) is a neurodevelopmental disorder typically appearing before the age of three and potentially persisting into adulthood. It is primarily characterized by social impairments, sensory processing issues, and repetitive behavior patterns, which may also result in obesity (1), sleep disturbances, motor abnormalities and injuries (2, 3), and executive function disorders (4). According to the World Health Organization (WHO), children and adolescents aged 3–16 are the most affected by autism, with approximately 1 in 100 children diagnosed with the disorder (5), and the prevalence is rising. The poor prognosis and high disability rate of children with autism impose significant mental and economic burdens on society and families (6). Currently, there is no cure for autism, and the causes and mechanisms of the disorder are not fully understood. Factors such as genetics, parental age, maternal mental health during pregnancy, diet, and children's metal metabolism disorders are considered risk factors (7).

In clinical practice, treatment for children with ASD mainly includes pharmacological and various non-pharmacological interventions. Pharmacological treatments target brain function, Attention Deficit Hyperactivity Disorder (ADHD), and depression symptoms in children with autism (8), but not autism itself. Long-term medication use can cause adverse effects such as weight gain, sedation, and extrapyramidal symptoms (9). Non-pharmacological treatments typically include speech therapy (10), psychosocial therapy (11), and behavioral interventions (12). However, these measures require early intervention (13) and significant time, effort, and financial investment. Scientifically and efficiently intervening with children with ASD to aid their growth and recovery is a pressing issue in today's society.

In recent years, physical exercise has gained attention and recognition as an intervention for children with autism. Studies have shown that physical activity can improve social (14), communication (14, 15), and motor skills in children with autism. Other studies have indicated that individual sports such as swimming (16) and horseback riding (17), as well as team sports like basketball (18) and soccer (19), can effectively reduce shyness, and enhance physical fitness, social skills, and communication abilities in children with autism (20). Randomized controlled trials (RCTs) have shown that, after ten weeks of instruction in gross motor skills and object control skills, children with autism significantly improved in overall gross motor skills, object control skills, and physical mobility (21).

Although many RCTs have studied the efficacy of exercise interventions on flexibility and cognitive control, social skills, behavioral problems, motor skills, and coordination in children with autism, the results are inconsistent. Some studies have shown that water sports interventions in the experimental group did not significantly improve social skills in seven children with ASD (22). Additionally, a meta-analysis by Shimeng Wang et al. included non-randomized controlled trials (23), and a systematic review by Qun Fang et al. included only one randomized controlled trial among ten studies (24). Some systematic reviews and meta-analyses only covered social skills and motor skills without addressing cognitive control and behavioral problems.

Therefore, this study collects RCTs on exercise interventions for children with autism and conducts a systematic review and meta-analysis to summarize the effects of different exercise modalities on flexibility and cognitive control, social skills, behavioral problems, motor skills, and coordination in children with autism. The purpose is to provide evidence-based medical guidance for prescribing appropriate exercise regimens for children with autism.

## Research methods

This meta-analysis involved randomized controlled trials and comparisons of physical exercise interventions on social, behavioral, and motor skills in children with autism. The protocol for this systematic review was retrospectively registered with INPLASY (INPLASY202510046).

### Search strategy

Five major databases were selected in this study—PubMed, EMbase, Cochrane Library, EBSCOhost, and Web of Science—for literature retrieval to ensure comprehensiveness and authority of the search results. The timeframe for the literature search was set from the establishment of the databases to February 15, 2024. This timeframe was chosen to cover all relevant studies and provide the latest and most comprehensive literature.

Search terms were determined through Mesh headings, focusing primarily on two categories: T1 (autism-related terms) and T2 (exercise-related terms). For T1 included Autistic Disorder, Autism, Infantile Autism, and Kanner's Syndrome. For T2 included Exercises, Physical Activity, Activities, Physical, Acute Exercise, Isometric Exercise, Aerobic Exercise, and Exercise Training. In the literature search, P (Population) was children with autism, I (Intervention) was exercise, C (Comparison) was no exercise intervention (no restrictions during search), O (Outcome) was autism-related indicators (such as the Social Communication Questionnaire, Childhood Autism Rating Scale, Autism Behavior Checklist, Social Responsiveness Scale, Autism Diagnostic Observation Schedule, etc., no restrictions during search), and S (Study design) was randomized controlled trials (RCT, no restrictions during search). The search language was English to ensure accuracy and consistency of the search results (Table 1).

**Table 1:** Search terms.

ID	Subject terms	Mesh terms
T1	Autistic disorder	Disorder, Autistic; Disorders, Autistic; Kanner's Syndrome Kanner Syndrome; Kanners Syndrome; Autism, Infantile Infantile Autism; Autism, Early Infantile; Early Infantile Autism; Infantile Autism, Early
T2	Exercises	Exercises OR Physical Activity OR Activities, Physical OR Activity, Physical OR Physical Activities OR Exercise, Physical OR Exercises, Physical OR Physical Exercise OR Physical Exercises OR Acute Exercise OR Acute Exercises OR Exercise, Acute OR Exercises, Acute OR Exercise, Isometric OR Exercises, Isometric OR Isometric Exercises OR Isometric Exercise OR Exercise, Aerobic OR Aerobic Exercise OR Aerobic Exercises OR Exercises, Aerobic OR Exercise Training OR Exercise Trainings OR Training, Exercise OR Trainings, Exercise
T3	T1 And T2	
Search language: English		Search Date: February 15, 2024

### Inclusion and exclusion criteria

#### Inclusion criteria

The literature included in this study must meet the following criteria:

1. The subjects must be children diagnosed with ASD to ensure the homogeneity of the study population and the comparability of results.
2. The intervention must be any form of exercise, including but not limited to aerobic exercise, strength training, and coordination training, to assess the effects of different types of exercise interventions on children with ASD.
3. The study must report at least one autism-related outcome measure, such as the Social Communication Questionnaire, Childhood Autism Rating Scale, Autism Behavior Checklist, Social Responsiveness Scale, and Autism Diagnostic Observation Schedule, to ensure the comprehensiveness and relevance of outcome measures.
4. The study design must be a RCT or quasi-experimental study to ensure the scientific validity and reliability of the research design and results.

#### Exclusion criteria

The excluded studies include the following:

1. Non-original research, such as reviews, conference abstracts, and commentary articles.
2. Studies involving adults or non-ASD children as subjects, as these do not meet the target population of this study.
3. Studies that do not provide sufficient statistical data for meta-analysis. The exclusion of these studies aims to improve the precision and validity of the analysis results.

### Data extraction and quality assessment

#### Data extraction process

After literature screening, two independent researchers extracted data from the included studies. Data extraction used standardized forms designed by the Cochrane Handbook to ensure systematic and consistent data collection. Researchers read the full texts and filled out the data extraction forms, recording all relevant information. If discrepancies arose during data extraction, a third party made the final decision to ensure data accuracy and consistency. The extracted data covered various aspects of the studies, including authors, publication years, study designs, sample sizes, types of interventions, intervention durations, and primary outcome measures. These measures included social communication abilities, flexibility, cognitive control, motor skills, coordination, and behavioral issues. By thoroughly documenting these data points, we could comprehensively assess the impact of exercise interventions on children with ASD.

### Quality assessment

To assess the quality of the included studies, this study used the Physiotherapy Evidence Database (PEDro) scale. The PEDro scale is a validated quality assessment tool widely used in evaluating the quality of randomized controlled trials in the field of physical therapy, with high reliability and validity. The quality assessment included multiple dimensions: random allocation, blinding, baseline comparability, intervention descriptions, reliability of outcome measures, and completeness of follow-up. Each study was scored based on these dimensions, with higher scores indicating higher quality. Through quality assessment, we could determine the risk of bias in the included studies, ensuring the reliability and scientific validity of the meta-analysis results.

### Effect size calculation

In this study, effect sizes were calculated using standardized mean differences (SMD) to measure the impact of exercise interventions on children with ASD. The use of SMD allows comparison of results across different studies employing various measurement tools, ensuring comparability. The 95% confidence interval (CI) for each effect size was also calculated to provide a precise range for the effect size. By calculating SMD and 95% CI, we could quantify the overall effect of exercise interventions across different studies and assess their statistical significance.

To evaluate heterogeneity among the included studies, we used the *I*^2^ statistic. The *I*^2^ value quantifies the proportion of total variation due to heterogeneity. An *I*^2^ value exceeding 50% indicates moderate to high heterogeneity. In such cases, we used a random effects model for analysis to account for variability among studies. If the *I*^2^ value was below 50%, indicating low heterogeneity, we used a fixed effects model for analysis. This approach allowed us to accurately assess the impact of exercise interventions on children with ASD while considering differences among studies.

#### Subgroup analysis

To gain a deeper understanding of the impact of exercise interventions on different subgroups, we conducted multiple subgroup analyses. Participants were grouped by grade level (preschool children, lower grade students, middle grade students, and upper grade students) to evaluate differences in responses to exercise interventions among different grade levels. Interventions were also grouped by type (aerobic exercise, strength training, coordination training, ball sports, physical training, martial arts, etc.) to identify the effects of different types of exercise interventions. Participants were further grouped by severity of autism (mild, moderate, and severe) to assess responses to interventions among different levels of autism severity. Interventions were also grouped by duration (short-term <8 weeks, mid-term 8–12 weeks, long-term >12 weeks) to determine the impact of intervention duration on outcomes. Through these subgroup analyses, we identified differences in effects under specific conditions, providing more targeted intervention strategies.

#### Publication bias analysis

To evaluate the presence of publication bias in the included studies, we utilized Begg's Test and Egger's Test, as they provide complementary perspectives. Begg's Test, based on rank correlation analysis (25), is particularly suitable for small sample sizes, while Egger's Test, which employs regression analysis, is more sensitive to detecting subtle publication bias (26). The combination of these two methods offers a comprehensive assessment of bias risk and enhances the robustness of our findings, as their effectiveness and reliability have been validated in multiple studies (27, 28). The funnel plot was used for qualitative analysis, further strengthening the credibility of our results. This integrated approach ensures a thorough and reliable evaluation of publication bias.

#### Sensitivity analysis

To verify the robustness of the results, we conducted sensitivity analysis on all included studies. The specific method involved sequentially excluding each study and re-analyzing the data to observe changes in effect sizes. This process aimed to assess the impact of individual studies on the overall results and detect the robustness of the findings. By using this exclusion method, if the effect sizes and conclusions remained consistent, it indicated high robustness and reliability of the study results. This method helped validate the consistency of research conclusions under different conditions, ensuring the final conclusions' credibility.

## Research results

### Literature search and screening process

**Figure 1:**
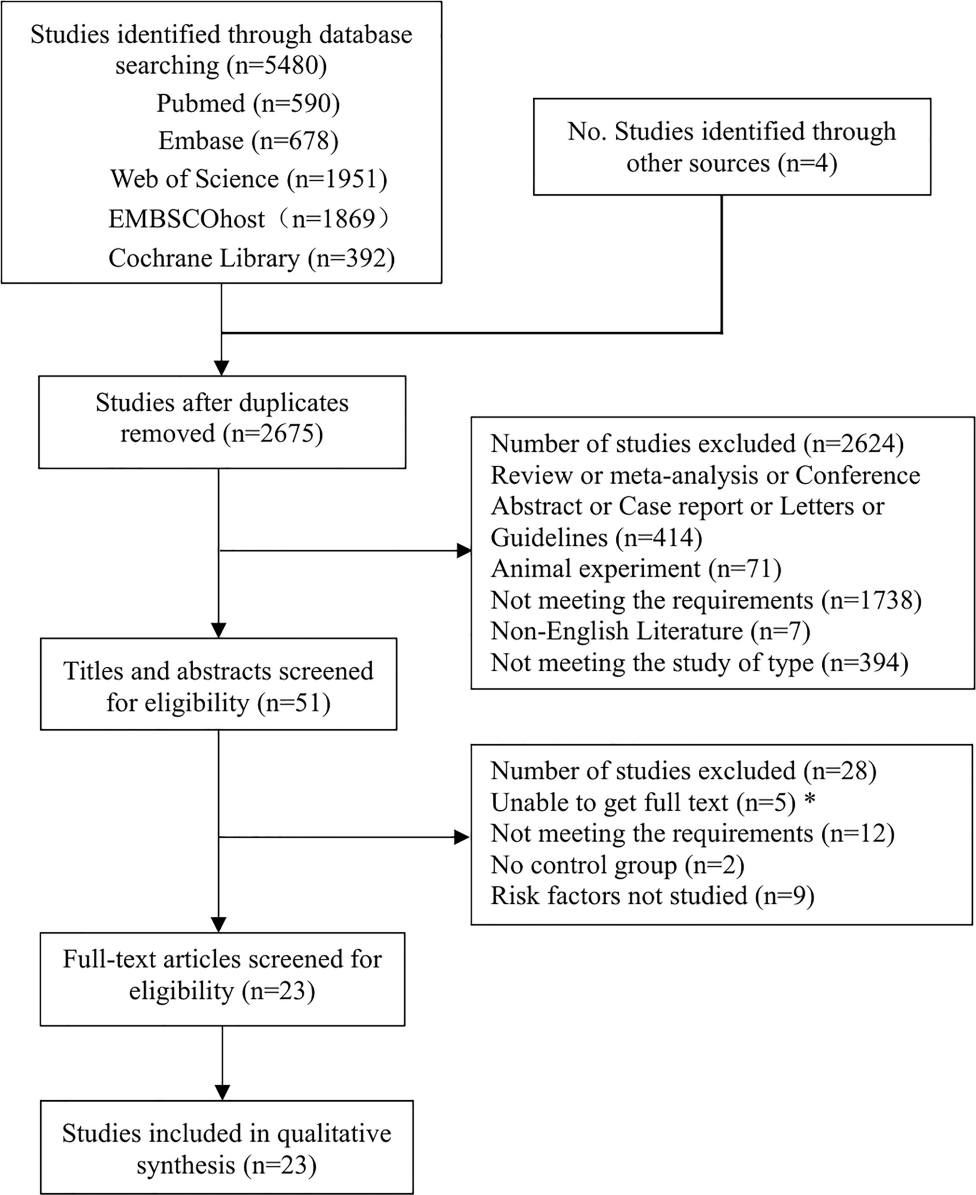
Flow diagram of study selection process.

This study retrieved a total of 5,480 articles from multiple databases, including Pubmed, Embase, Web of Science, EBSCO host, and Cochrane Library, and identified 4 additional articles through other sources (reference tracking). After removing duplicates using EndnotesX20, 2,675 articles remained. At the title and abstract screening stage, 2,624 articles were excluded for reasons such as reviews or meta-analyses, conference abstracts, case reports, letters or guidelines, animal experiments, not meeting requirements, non-English articles, and non-relevant study types. Subsequently, 51 articles underwent full-text review, and 28 articles were excluded based on the inclusion and exclusion criteria due to reasons such as unavailability of full text, not meeting requirements, lack of control groups, and not studying risk factors. Finally, 23 articles were included for meta-analysis (Figure 1).

### Characteristics of included studies

The studies included in this research involved intervention studies on children with ASD and assessed multiple key indicators. These indicators included gender, age, sample size, intervention type, autism status, intervention duration, and control group intervention methods (Table 2). The intervention and control groups in the studies included both boys and girls, with roughly matched gender ratios to ensure the scientific validity and reliability of the results. The age of the subjects ranged from preschool children to adolescents, covering different developmental stages of children with autism. Sample sizes varied from as few as 13 to as many as 148, ensuring data diversity and representativeness. The studies detailed the specific conditions of children with autism, including mild intellectual disability, preschool autism, and anxiety-related autism, with some studies referencing standardized diagnostic tools (ADOS-2 and DSM-5).

**Table 2:** Characteristics of included studies.

Author	Year	Gender	Age	*N*	ID	Intervention method	Autism status	Intervention period	Control group intervention method
Andy	2023	Exc1: 20B3G, Exc2: 13B6G, Con: 19B3G	Exc1: 9.61 ± 1.41, Exc2: 10.16 ± 1.38, Con: 10.275 ± 1.39	64	1	Ride a bicycle	mild intellectual disability	2weeks	Walking
Hao Chen	2024	Exc: 12B3G, Con: 10B5G	Exc: 5.27 ± 0.70, Con: 5.07 ± 1.10	30	2	Play sport games	Age range of 3 to 6 years and an IQ ≥ 70; Absence of hearing and visual impairments;	8weeks	Maintain original rehabilitation plan
AdrianaKaplánová	2022	Exc: 9B1G, Con: 8B2G	Exc: 6.78 ± 1.34, Con: 8.25 ± 1.44	20	4	Use the TGMD-2 to improve locomotion	ADOS-2 Autism Diagnostic Observation	8weeks	Maintain original rehabilitation plan
Yu-Ru Jin	2023	Exc: 7B, Con: 6B	mean age 4.91 years	13	5	Eight 90-min sports sessions	–	8weeks	Maintain original rehabilitation plan
Amir Hossein Haghighi1	2023	Exc: 5B3G, Con: 4B4G	Exc: 9.00 ± 1.31, Con: 8.13 ± 1.36	16	7	Combined physical training (CPT)	–	8weeks (three sessions per week).	10 minutes of stationary cycling, 5 minutes warm-up and cool-down
MUHAMMAD SAAD SHAFIQ	2022	26B, 4G	mean age: 12.73 ± 1.62	30	13	Perform stationary cycling	Autistic child	5weeks	LEGO/Minecraft
Jean-G. Gehricke	2022	Exc: 84%B, Con: 83%B	Exc: 9.3(2.0), Con: 9.7(2.2)	148	15	Physical exercise	anxious ASD children	8weeks	Walking
Sixin Yang	2021	Exc: 12B3G, Con: 13B2G	Preschool children	30	18	12-week mini-basketball training program	preschool children with autism spectrum disorders	12weeks	Only receive routine behavioral rehabilitation
ANDY C. Y. TSE	2021	50B12G	Mage: 9.89 ± 1.53	62	19	Learning to ride a bicycle, stationary cycling	general children with ASD	2weeks	Not cycling
Janice N. Phung	2021	Exc: 14B, Con: 14B6G	Exc: 9.10 ± 1.10, Con: 9.10 ± 1.10	34	21	Mixed Martial Arts (MMA)	mild intellectual disability	13weeks	Not participating in martial arts activities
Chinmoyee Nanda Panigrahy	2021	Exc: 6B4G, Con: 7B3G	Exc: 13.2 ± 2.7, Con: 14.1 ± 2.9	20	22	Bimanual exercises	children with autism spectrum disorder	6 months	Normal treatment
Soleyman Ansari1	2020	Exc1: 10B, Exc2: 10B, Con: 10B	Exc1: 10.60 ± 2.50,Exc2: 10.80 ± 2.14, Con: 10.80 ± 2.44	30	25	Land-based and swimming-based exercise	ASD from Guilan Autism Society Institute	10weeks	No intervention
Jin-Gui Wang	2020	Exc: 15B3G, Con: 13B2G	Exc: 5.11 ± 0.65, Con: 4.70 ± 0.70	33	27	Mini-basketball training program	meeting the Diagnostic and Statistical Manual of Mental Disorders, 5th edition, criteria for ASD	12weeks	Maintain daily activities
Ke-Long Cai	2020	Exc: 14B1G, Con: 12B3G	Exc: 4.56 ± 0.84, Con: 5.03 ± 0.64	59	30	Mini-basketball training program	Childhood Autism Rating Scale 2	12weeks	No intervention
Kelong Cai	2020	Exc: 13B1G, Con: 12B3G	Exc: 4.68 ± 0.72, Con: 5.13 ± 0.61	29	31	Mini-basketball training	ASD via DSM-5	12weeks	No intervention
Choi Yeung Andy Tse	2019	Exc: 14B5G, Con: 18B3G	Exc: 10.11 ± 1.20, Con: 9.81 ± 1.17	40	32	Exercises	–	12weeks	No intervention
Wenxin Xu	2018	Exc: 40B12G, Con: 42B12G	Exc: 14.8 ± 6.1, Con: 15.5 ± 5.1	106	35	Exercise-based rehabilitation model	–	4months	No intervention
Chien-Yu Pan	2016	Exc: 11B, Con: 11B	Exc: 10.3 ± 2.1, Con: 11.2 ± 1.9	22	40	Physical activity	a diagnosis of ASD based on Diagnostic and Statistical Manual of Mental Disorders, 4th edition	12weeks	No intervention
Fatimah Bahrami	2016	Exc: 13B2G, Con: 13B2G	Exc: 9.20 ± 3.32, Con: 9.06 ± 3.33	30	43	Karate techniques training	Autism severity 42.53 ± 18.65	14weeks	No intervention
Carla Lourenço	2015	Exc: 1B5G, Con: 4B7G	Exc: 5.125 ± 1.552, Con: 8 ± 2.190	17	44	Trampoline training	mild/moderate ASD, have a lower engine performance	20weeks	Not participating in exercise
Agnes S. Chan	2013	Exc: 19B1G, Con: 17B3G	Exc: 11.28 ± 3.90, Con: 12.42 ± 3.25	40	49	Nei Yang Gong (traditional Chinese mind-body exercise)		4weeks	No intervention
Fatimah Bahrami	2012	Exc: 13B2G, Con: 13B2G	Exc: 9.20 ± 3.32, Con: 9.06 ± 3.33	30	50	Kata techniques training	Autism severity 42.53 ± 18.65	14weeks	Not participating in exercise
Margaret M. Bass Æ	2009	Exc: 2B17G, Con: 3B12G	Exc: 6.95 ± 1.67, Con: 7.73 ± 1.65	34	51	Therapeutic horseback riding	meet criteria for DSM-IV-TR(American Psy-chiatric Association 2000)	12weeks	Not participating in exercise

The intervention methods were diverse, including cycling, physical games, motor skill training, bilateral training, karate, trampoline training, stationary cycling, and comprehensive physical training, aiming to improve the motor skills and social abilities of children with ASD through various physical and sports activities. The intervention duration ranged from two weeks to six months, with most studies having an intervention period of eight to twelve weeks, ensuring sufficient time to observe intervention effects. Control groups typically maintained regular treatment or did not receive additional interventions, serving as a baseline for comparison. Some control groups continued with their existing rehabilitation plans without physical activity, while others engaged in routine daily activities or no interventions at all. This setup helped assess the actual effects of the interventions, supporting the effectiveness of physical activity as a rehabilitation means for children with ASD through these systematic intervention studies.

### Quality assessment of included studies

All included studies met the inclusion criteria (Table 3) and employed random allocation, but there were some shortcomings in allocation concealment, with only a few studies using allocation concealment measures. Most studies ensured comparability of subjects at baseline. Regarding blinding, almost all studies did not blind subjects and therapists, with only a few studies blinding assessors. Most studies maintained an appropriately low attrition rate (<15%) during follow-up and conducted intention-to-treat analysis. In terms of intergroup comparison, most studies performed appropriate analyses and reported specific effect sizes and variability indicators. The PEDro scale scores indicated that the highest-scoring study achieved 9/10, reflecting good performance across various quality indicators, while the lowest-scoring study achieved 5/10, indicating deficiencies in several key quality indicators.

**Table 3:** Methodological quality evaluations of included studies.

Serial number	Author	Year	Eligibility criteria	Random allocation	Concealed allocation	Baseline similarity	Blind subjects	Blind therapists	Blind assessor	Adequate follow-up dropout <15%	Intention-to-treat analysis	Between-group comparisons	Point and variability measures	Total score
1	Andy	2023	1	1	0	1	0	0	0	1	1	1	1	6/10
2	Hao Chen	2024	1	1	1	1	0	0	0	1	1	1	1	7/10
4	AdrianaKaplánová	2022	1	0	0	1	0	0	0	1	1	1	1	5/10
5	Yu-Ru Jin	2023	1	1	1	1	1	1	1	1	0	1	1	9/10
7	Amir Hossein Haghighi1	2023	1	1	1	1	0	0	1	1	1	1	1	7/10
13	MUHAMMAD SAAD SHAFIQ	2022	1	1	0	1	0	0	0	1	1	1	1	6/10
15	Jean-G. Gehricke	2022	1	1	0	1	0	0	1	1	1	1	1	7/10
18	Sixin Yang	2021	1	1	0	1	0	0	1	1	1	1	1	7/10
19	ANDY C. Y. TSE	2021	1	1	0	1	0	0	1	1	0	1	1	6/10
21	Janice N. Phung	2021	1	1	0	1	0	0	1	1	1	1	1	7/10
22	Chinmoyee Nanda Panigrahy	2021	1	1	0	1	0	0	0	1	1	1	1	6/10
25	Soleyman Ansari1	2020	1	1	0	1	0	0	0	1	1	1	1	6/10
27	Jin-Gui Wang	2020	1	0	0	1	0	0	0	1	1	1	1	5/10
30	Ke-Long Cai	2020	1	1	0	1	0	0	0	0	1	1	1	5/10
31	Kelong Cai	2020	1	1	0	1	0	0	0	0	1	1	1	5/10
32	Choi Yeung Andy Tse	2019	1	1	0	1	0	0	1	1	0	1	1	6/10
35	Wenxin Xu	2018	1	1	0	1	0	0	0	1	0	1	1	5/10
40	Chien-Yu Pan	2016	1	1	0	1	0	0	0	1	0	1	1	5/10
43	Fatimah Bahrami	2016	1	1	0	1	0	0	0	1	1	1	1	6/10
44	Carla Lourenço	2015	1	1	0	1	0	0	0	1	1	1	1	6/10
49	Agnes S. Chan	2013	1	1	0	1	0	0	0	1	1	1	1	6/10
50	Fatimah Bahrami	2012	1	1	0	1	0	0	0	1	0	1	1	5/10
51	Margaret M. Bass Æ	2009	1	1	0	1	0	0	0	1	0	1	1	5/10

The included studies performed well in random allocation, baseline similarity, low attrition rate, and intergroup comparison, but there is room for improvement in allocation concealment and blinding. The quality assessment using the PEDro scale provided a reliable basis for this meta-analysis and highlighted areas for improvement in the design and implementation of future studies.

### Meta-analysis results

#### Impact of exercise on flexibility and cognitive control in children with ASD

**Figure 2:**
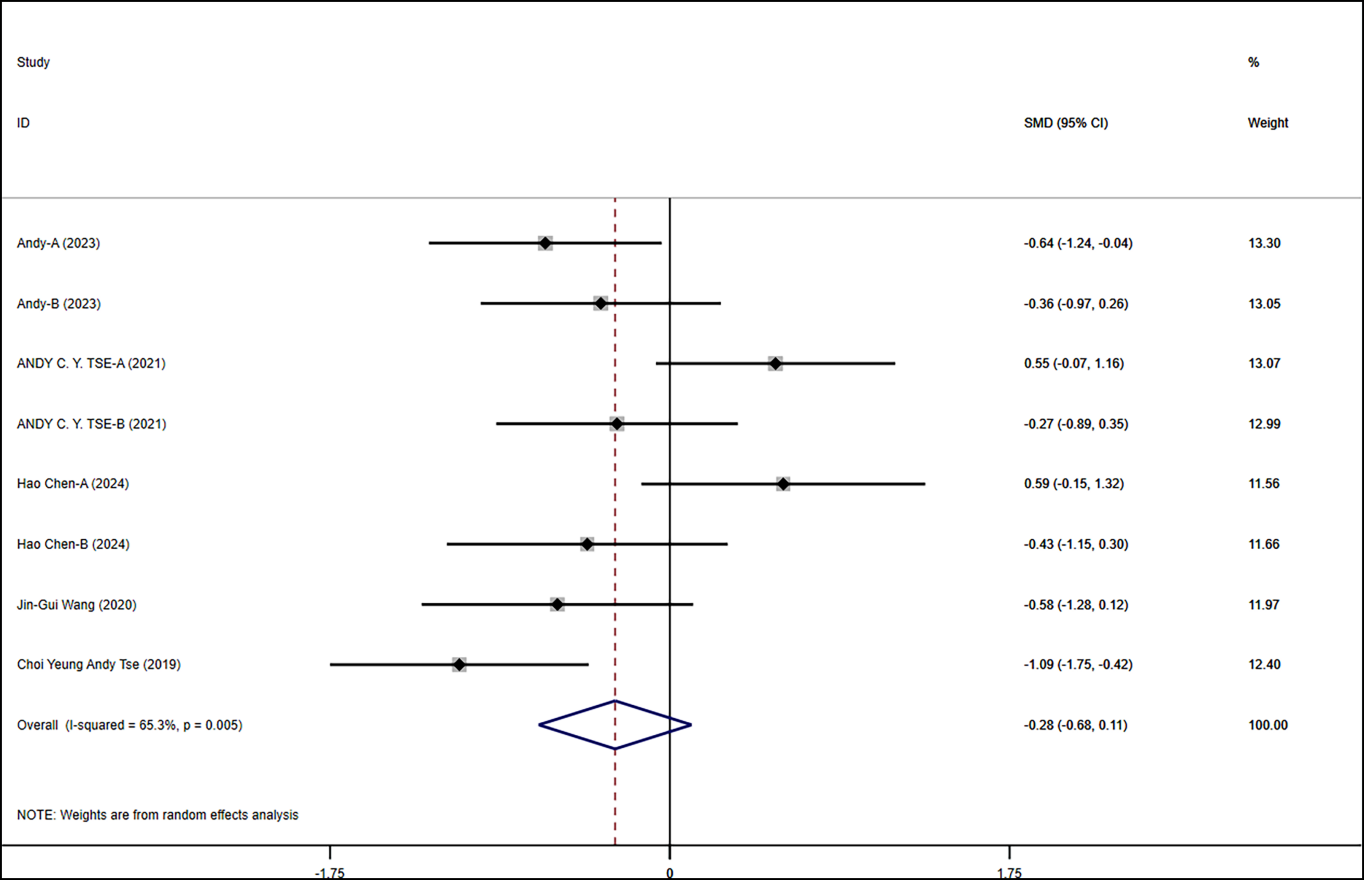
Meta-analysis of exercises on flexibility and cognitive.

This study examined the impact of exercise on flexibility and cognitive control in children with ASD. Overall results indicated that exercise interventions had positive effects on flexibility and cognitive control in children with ASD (SMD = −0.282, 95% CI = −0.676, 0.112, *p* = 0.161). Although the overall effect did not reach statistical significance, the results still suggested that exercise interventions had positive effects on flexibility and cognitive control across multiple studies (Figure 2).

**Figure 3:**
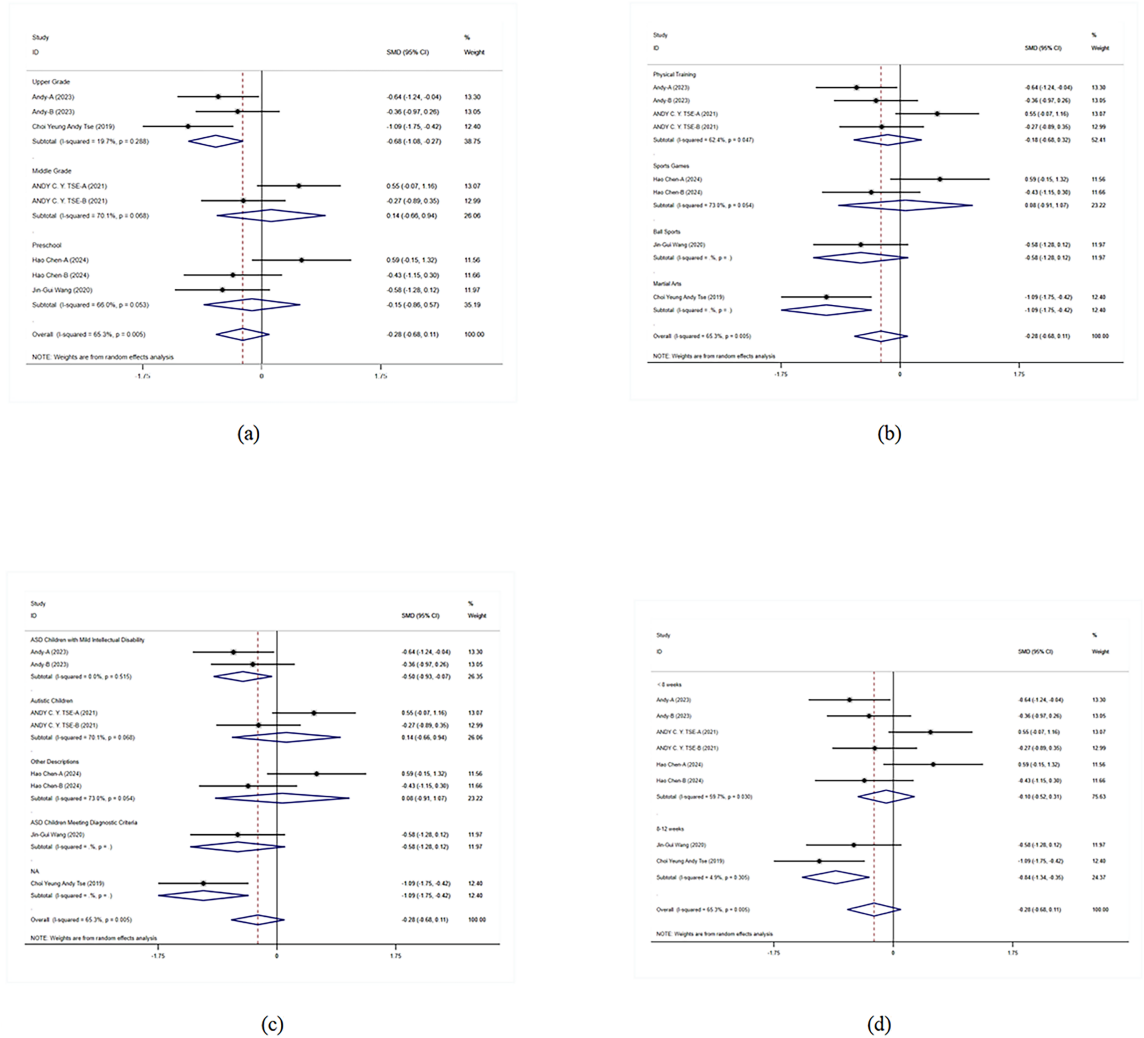
Subgroup analysis of the effect of exercise on flexibility and cognitive control in children with ASD. **(a)** Flexibility and cognitive control grade subgroup analysis. **(b)** Flexibility and cognitive control intervention type subgroup analysis. **(c)** Flexibility and cognitive control disease type subgroup analysis. **(d)** Flexibility and cognitive control intervention duration subgroup analysis.

In the grade-level subgroup analysis, upper-grade students showed significant improvement, indicating marked enhancement in flexibility and cognitive control. In contrast, the effects in middle-grade students and preschool children were not statistically significant, with higher heterogeneity observed. Upper-grade students responded more consistently to exercise interventions, while there was greater variability in effects among other grades. Therefore, designing specific interventions for different grade levels may help improve overall intervention effectiveness. In the subgroup analysis of intervention forms and autism severity, physical training and exercise games did not show significant effects on flexibility and cognitive control in children with ASD, whereas martial arts interventions had significant positive effects. Moderate autism severity showed significant intervention effects, while effects in other autism severities were not evident. Analysis of intervention duration effects indicated that interventions lasting 8–12 weeks had significant effects, suggesting that an appropriate duration of exercise intervention could more effectively improve flexibility and cognitive control in children with ASD. These results emphasized the importance of intervention forms and duration, indicating that adjusting intervention strategies to meet specific needs is crucial for improving intervention outcomes (Figure 3).

#### The impact of exercise on motor skills and coordination in children with ASD

**Figure 4:**
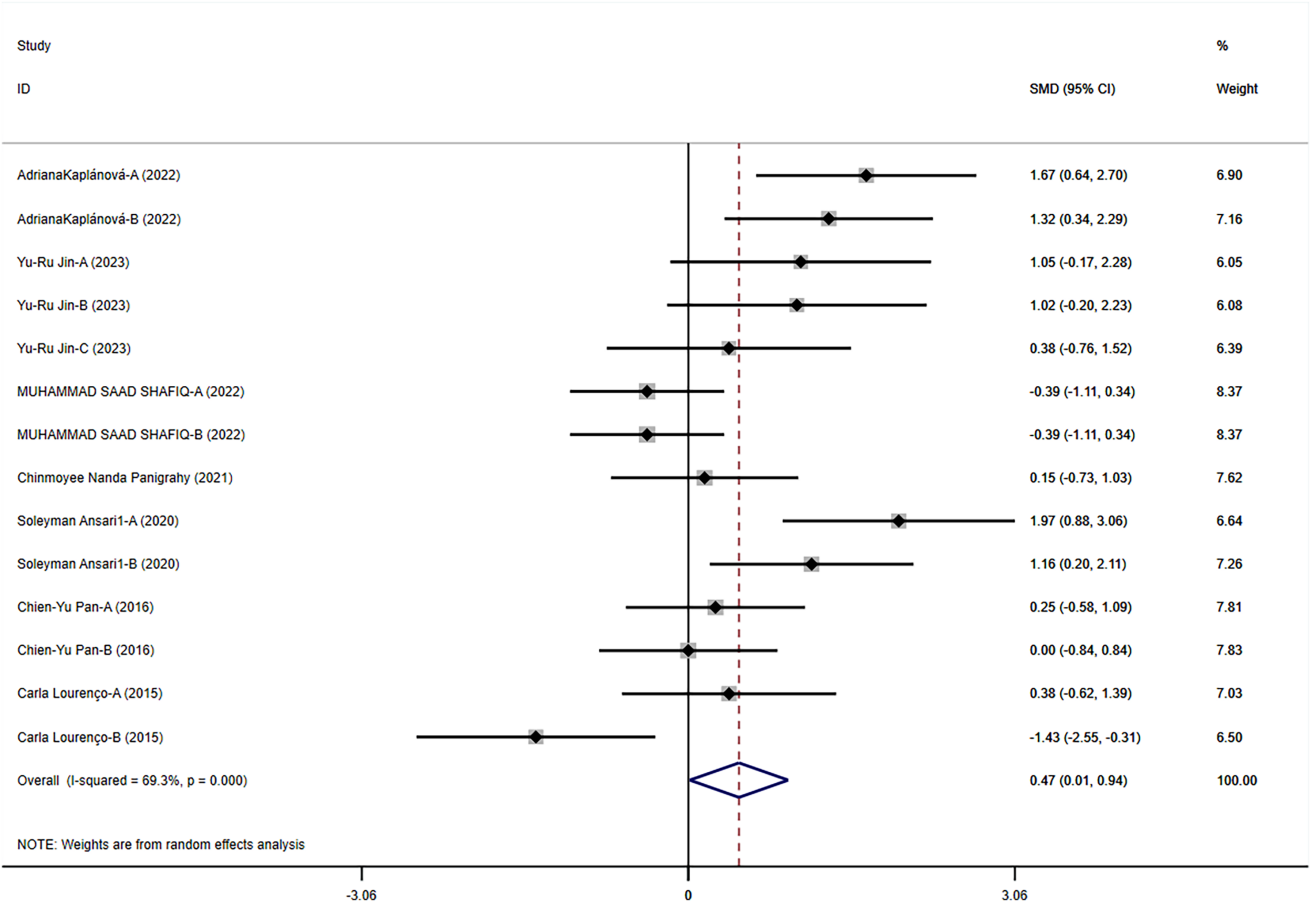
Meta-analysis of exercises on motor skills and coordination.

The results of this meta-analysis show that exercise interventions have a significant positive effect on motor skills and coordination in children with ASD (SMD = 0.475, 95% CI = 0.014–0.936, *p* = 0.043, *I*^2^ = 69.30%). This indicates that exercise interventions positively influence motor skills and coordination in children with ASD across multiple studies (Figure 4).

**Figure 5:**
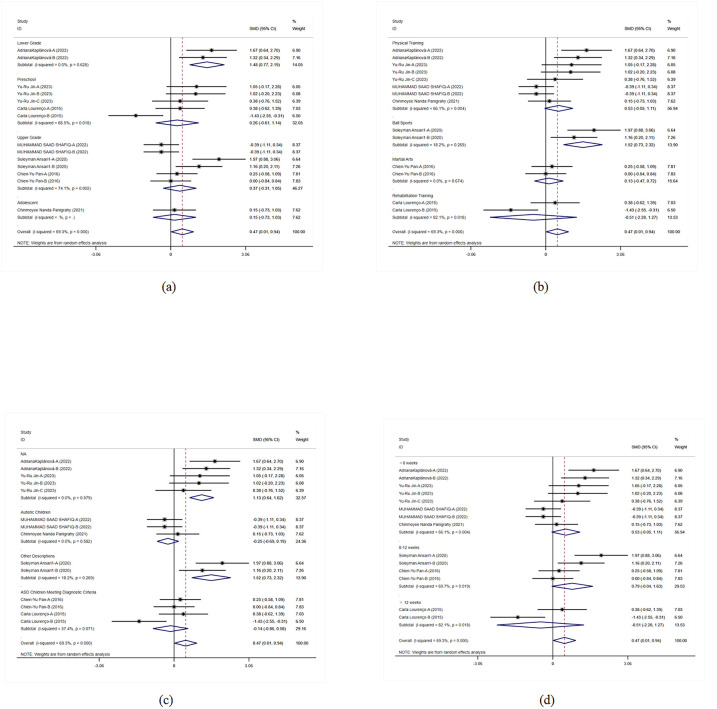
Subgroup analysis of the effect of exercise on motor skills and coordination in children with ASD. **(a)** Motor skills and coordination grade subgroup analysis. **(b)** Motor skills and coordination intervention type subgroup analysis. **(c)** Motor skills and coordination disease type subgroup analysis. **(d)** Motor skills and coordination intervention duration subgroup analysis.

In the grade-level subgroup analysis, the effects of exercise interventions were most pronounced in lower-grade students, showing significant improvements (SMD = 1.482, *p <* 0.001). The intervention effects were not significant for preschool children and upper-grade students, and heterogeneity was high; the intervention effects in adolescents also did not reach significant levels. This suggests that lower-grade students respond more consistently to exercise interventions, while there is greater variability in the effects on other grade levels. Therefore, designing specific interventions for different grade levels may help improve overall intervention effectiveness. In the subgroup analysis of intervention forms and disease types, ball sports showed significant positive effects (SMD = 1.521, *p <* 0.001), whereas physical training and rehabilitation training did not reach significant levels. Martial arts interventions also did not show significant effects. Among disease types, children with unspecified ASD showed significant improvement, while those with ASD and those meeting specific ASD criteria did not show significant intervention effects. Analysis of intervention duration effects showed that short-term (<8 weeks) and mid-term (8–12 weeks) interventions had better effects, but interventions longer than 12 weeks did not reach significant levels (Figure 5).

#### The impact of exercise on social skills in children with ASD

**Figure 6:**
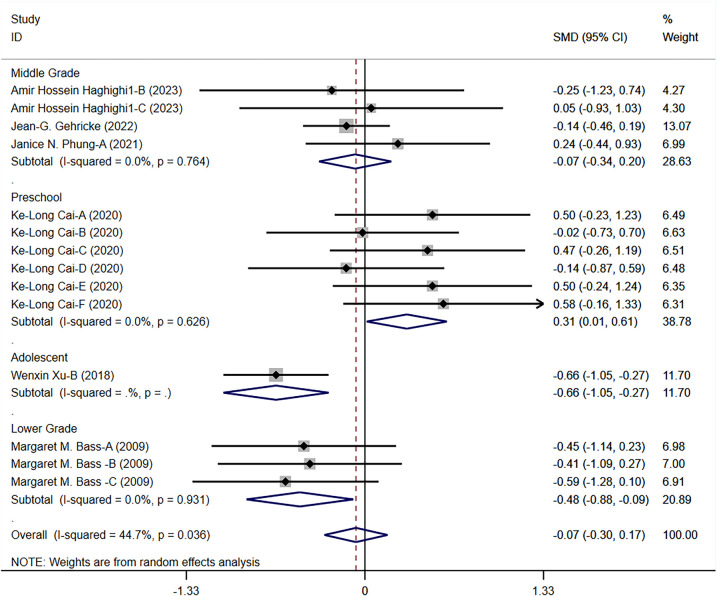
Forest plot of the effect of exercise on social skills in children with ASD.

The overall results of exercise interventions on social skills in children with ASD indicate that the effects on social skills were not significant (*p* = 0.578), with moderate heterogeneity (*I*^2^ = 44.70%) (Figure 6). This suggests that, overall, exercise interventions do not significantly improve social skills in children with ASD, necessitating further detailed analysis of specific intervention effects.

**Figure 7:**
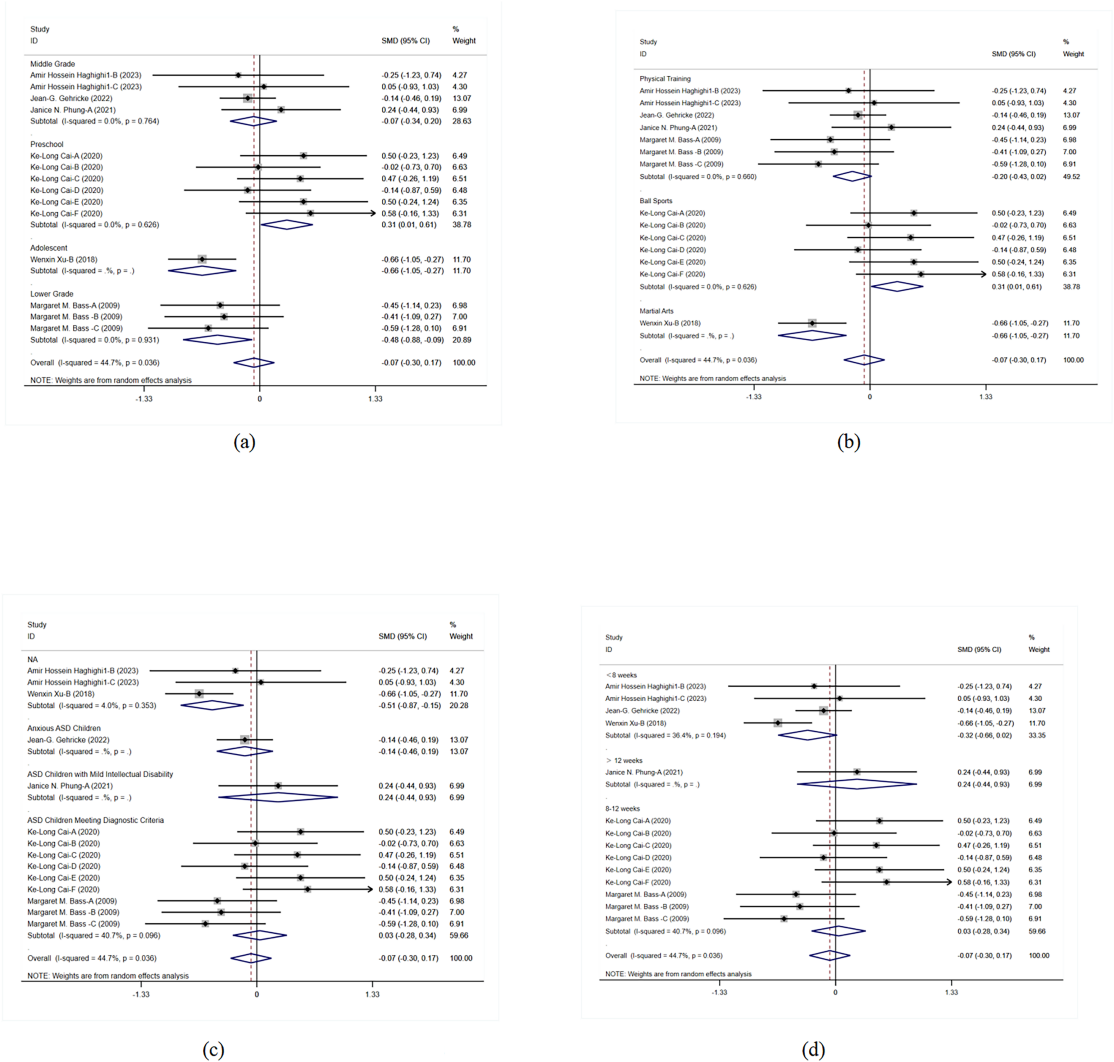
Subgroup analysis of the effect of exercise on social skills in children with ASD. **(a)** Social skills grade subgroup analysis. **(b)** Social skills intervention type subgroup analysis. **(c)** Social skills disease type subgroup analysis. **(d)** Social skills intervention duration subgroup analysis.

In the grade-level subgroup analysis, preschool children's social skills significantly improved (SMD = 0.312, 95% CI = 0.013–0.610, *p* = 0.041). The intervention effects in middle-grade and lower-grade students did not reach significant levels, although the effects in lower-grade students were close to significance (SMD = −0.484, *p* = 0.017). These results suggest significant variability in responses to exercise interventions among different grade levels, particularly between preschool children and adolescents. In the subgroup analysis of intervention forms, ball sports significantly improved social skills in children with ASD (SMD = 0.312, *p* = 0.041), whereas physical training and martial arts interventions did not show significant effects. In terms of disease types, interventions for children with anxiety-type ASD and those meeting specific ASD criteria did not show significant effects. The analysis of intervention duration effects showed that interventions of different durations did not reach significant levels (Figure 7).

#### The impact of exercise on behavioral problems in children with ASD

**Figure 8:**
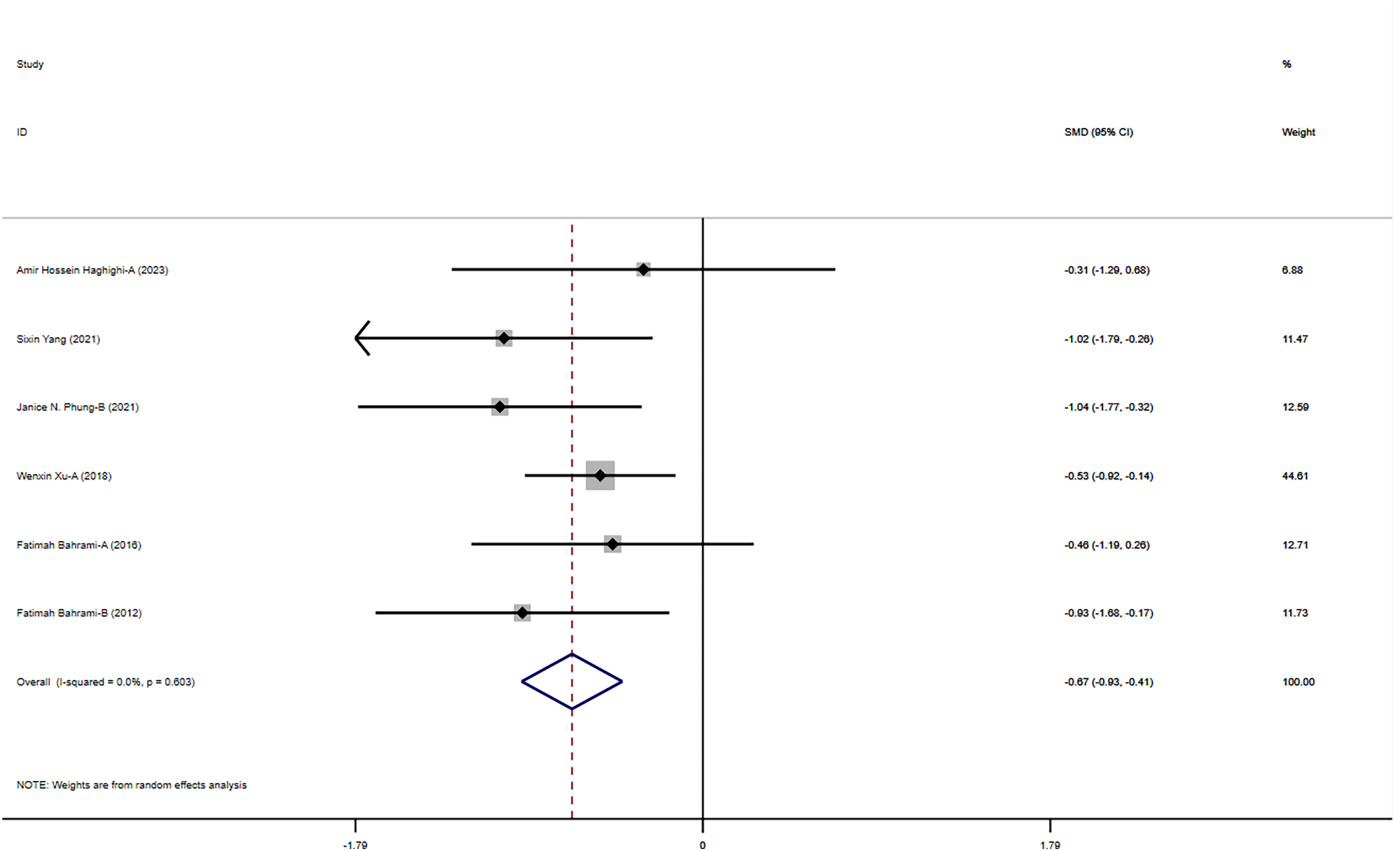
Meta-analysis of exercises on behavioral problems.

The meta-analysis results indicate that physical activity significantly improved behavioral problems in children with ASD (SMD = −0.674, 95% CI = −0.932 to −0.415, *I*^2^ = 0), showing consistent positive effects of various interventions across studies (Figure 8). This overall effect size indicates that the interventions have a significant positive impact in multiple studies, strongly supporting physical activity as an effective means to improve behavioral problems in children with ASD.

**Figure 9:**
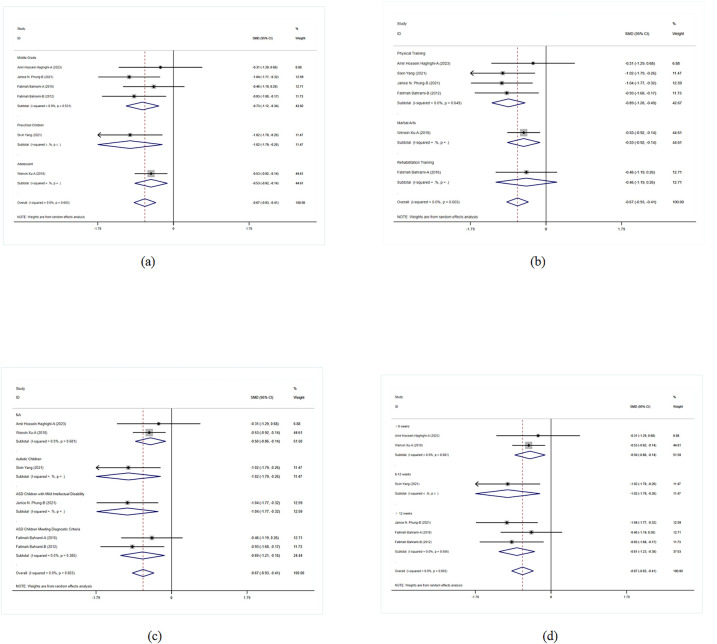
Subgroup analysis of the effect of exercise on behavioral problems in children with ASD. **(a)** Behavioral problems grade subgroup analysis. **(b)** Behavioral problems intervention type subgroup analysis. **(c)** Behavioral problems disease type subgroup analysis. **(d)** Behavioral problems intervention duration subgroup analysis.

Subgroup analysis results show significant improvements in middle-grade students and adolescents, with even more pronounced effects in preschool children. This indicates that the interventions have positive effects across different grade levels, particularly for preschool children. This finding supports the implementation of corresponding interventions at different grade levels to maximize their effects and ensure that children of all ages benefit. In the subgroup analysis of intervention forms and disease types, physical training and martial arts interventions showed significant effects, particularly in children with ASD and those with moderate ASD, while rehabilitation training had relatively weaker effects. The effects of different intervention durations were also significant, with interventions lasting 8–12 weeks showing particularly notable effects. This indicates that the form and duration of interventions significantly influence outcomes, necessitating the development of tailored intervention strategies based on specific needs to improve the effectiveness and specificity of the interventions (Figure 9).

#### Publication bias analysis

Publication bias analysis revealed no significant bias for flexibility and cognitive control indicators, as shown by both Begg's Test (z = 0.25) and Egger's Test (bias estimate = 1.63, 95% CI: −19.98 to 23.24), suggesting balanced data. However, for motor skills and coordination indicators, Egger's Test indicated a potential publication bias (bias estimate = 5.18, 95% CI: −0.35 to 10.70), corroborated by funnel plot analysis. While Begg's Test also suggested bias for these indicators, Egger's Test results reinforced this finding. For social skills indicators, Begg's Test results were near significance, but Egger's Test showed no significant bias. Similarly, both tests for behavioral problems indicators revealed no evidence of publication bias (Table 4).

**Table 4:** Begg's test and egger's test results.

Indicators	Test	Metric	Value	Test	slope	−0.8293092
Flexibility and cognitive control	Begg's test	adj. Kendall's Score (P-Q)	2	Egger's test	Std. Err.	2.950646
		Std. Dev. of Score	8.08		*t*	−0.28
		Number of Studies	8		P>	*t*
		*z*	0.25		95%CI	−8.04928, 6.390662
		Pr>	*z*		bias	1.63228
		*z* (continuity corrected)	0.12		Std. Err.	8.830572
		Pr>	*z*		*t*	0.18
					P>	*t*
					95%CI	−19.97535, 23.23991
Motor skills and coordination	Begg's test	adj. Kendall's Score (P-Q)	38	Egger's test	Std. Err.	1.217292
		Std. Dev. of Score	18.27		*t*	−1.71
		Number of Studies	14		P>	*t*
		*z*	2.08		95%CI	−4.729353, 0.5751485
		Pr>	*z*		bias	5.177096
		*z* (continuity corrected)	2.03		Std. Err.	2.535277
		Pr>	*z*		*t*	2.04
					P>	*t*
					95%CI	−0.3467974, 10.70099
Social skills	Begg's test	adj. Kendall's Score (P-Q)	33	Egger's test	slope	−0.6102981
		Std. Dev. of Score	18.27		Std. Err.	0.3154277
		Number of Studies	14		*t*	−1.93
		*z*	1.81		P>	*t*
		Pr>	*z*		95%CI	−1.297556, 0.0769599
		*z* (continuity corrected)	1.75		bias	1.646357
		Pr>	*z*		Std. Err.	1.017533
					*t*	1.62
					P>	*t*
					95%CI	−0.570657, 3.863372
Behavioral problems	Begg's test	adj. Kendall's Score (P-Q)	−3	Egger's test	Std. Err.	0.370726
		Std. Dev. of Score	5.32		*t*	−1.1
		Number of Studies	6		P>	*t*
		*z*	−0.56		95%CI	−1.436143, 0.6224577
		Pr>	*z*		bias	−0.8693462
		*z* (continuity corrected)	0.38		Std. Err.	1.146064
		Pr>	*z*		*t*	−0.76
		slope	−0.4068428		P>	*t*
					95%CI	−4.05133, 2.312638

**Figure 10:**
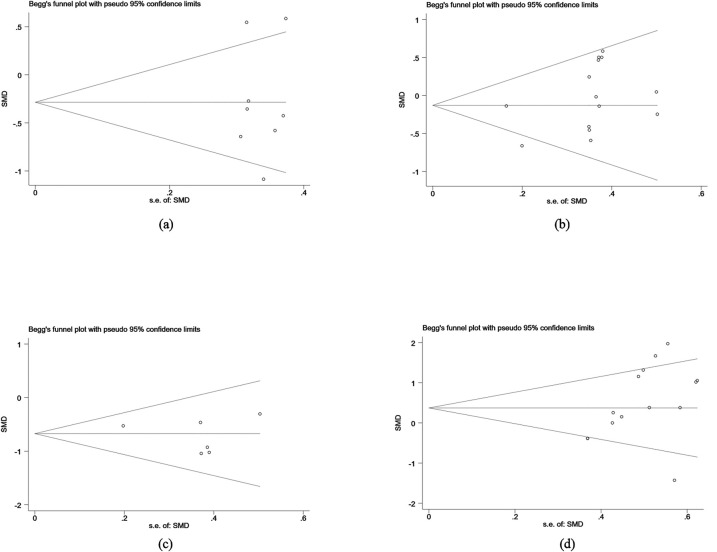
Begg's test. **(a)** Flexibility and cognitive control publication bias analysis. **(b)** Social skills publication bias analysis. **(c)** Behavioral problems publication bias analysis. **(d)** Motor skills and coordination publication bias analysis.

It can be seen that no significant publication bias was found in the analysis of flexibility and cognitive control, social skills, and behavioral problems indicators. However, motor skills and coordination indicators showed significant publication bias, indicating a certain risk of bias in this study, which needs further correction to improve the reliability and accuracy of the research results (Figure 10).

#### Sensitivity analysis

**Figure 11:**
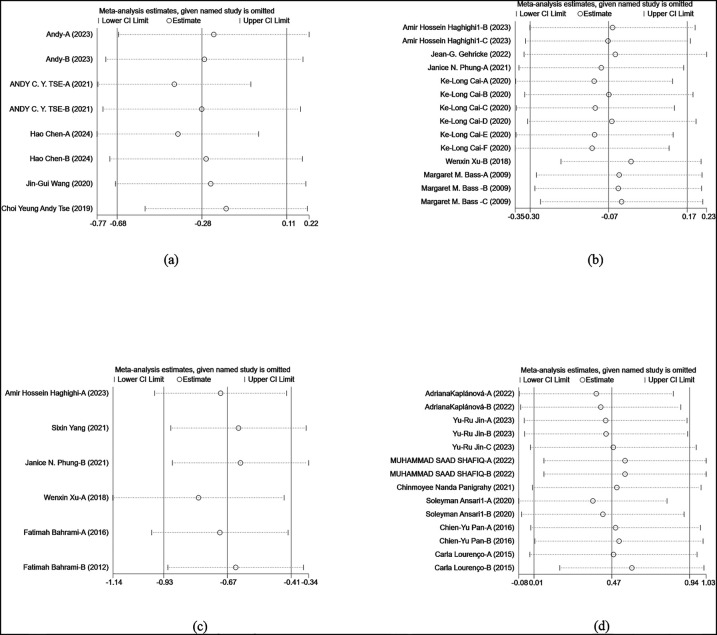
Sensitivity analysis. **(a)** Flexibility and cognitive control sensitivity analysis. **(b)** Social skills sensitivity analysis. **(c)** Behavioral problems sensitivity analysis. **(d)** Motor skills and coordination sensitivity analysis.

In the sensitivity analysis of flexibility and cognitive control, motor skills and coordination, social skills, and behavioral problems indicators, the results showed that the effect sizes did not change significantly after the sequential exclusion of studies, maintaining overall consistency in trends. This indicates the robustness of the research results. Even after excluding individual studies, the significance and direction of the results remained unchanged, further verifying the reliability of the conclusions. These analyses validated the reliability of the research conclusions, showing that the results remained consistent even under different exclusion conditions (Figure 11).

## Discussion

The potential of exercise interventions in improving the condition of children with ASD has garnered widespread attention. ASD is a complex neurodevelopmental disorder that affects children's social, communication, and behavioral abilities and is often accompanied by cognitive and motor function deficits. Enhancing flexibility and cognitive control is important for the daily lives and social interactions of children with ASD. In recent years, an increasing number of studies have explored the effects of various exercise interventions on different abilities of children with ASD, but the results have been inconsistent. This study found that exercise interventions positively impact the flexibility, cognitive control, motor skills, coordination, social abilities, and behavioral problems of children with ASD. Significant improvements were observed in upper and lower-grade students in their respective areas, preschool children's social abilities significantly enhanced, and behavioral problems improved across all grade levels. Martial arts and ball sports showed significant effects in various areas, and appropriate periods of exercise interventions can effectively enhance the abilities of children with ASD.

Regarding flexibility and cognitive control in children with ASD, although the overall effect in this study did not reach significance (SMD = −0.282, *p* = 0.161), exercise interventions showed positive effects in multiple studies. Previous research has indicated that exercise interventions can improve executive functions in children with ASD, including working memory and cognitive flexibility. A meta-analysis found that exercise interventions significantly improved working memory and inhibitory control in children with ASD (SMD = 0.28, *p* = 0.19; SMD = 1.30, *p* = 0.0004) (29). Another study found that 6–9 weeks of virtual training and physical exercise improved executive functions in children with ASD (30).

In terms of motor skills and coordination, this study found that exercise interventions had significant positive effects (SMD = 0.475, *p* = 0.043), especially in lower-grade students, where the effects were most pronounced (SMD = 1.482, *p <* 0.001). Similar results have been reported in other studies. Melek (2022) found that ball sports significantly improved motor skills and coordination in children with ASD (SMD = 1.521, *p <* 0.001) (31). Another study using square-stepping exercises found significant improvements in balance and cognitive skills in children with ASD (32). Regarding improvements in social skills and behavioral problems, this study showed that the overall effects were not significant (*p* = 0.578), but preschool children's social abilities significantly improved (SMD = 0.312, *p* = 0.041), and behavioral problems significantly improved across all grade levels (SMD = −0.674, *p <* 0.001). These findings are consistent with some studies that have indicated that specific exercise interventions, such as ball sports and martial arts, can significantly improve social skills and behavioral problems in children with ASD (29). However, other studies did not find significant effects, suggesting that the improvement in social skills may depend on the specific form and implementation of the intervention (30). Overall, the results of this study align with previous research, highlighting the positive impact of exercise interventions on various abilities of children with ASD, while also pointing out differences across different ages and intervention forms.

Systematic physical and coordination training can significantly improve flexibility and cognitive control in children with ASD. Repetitive physical activities promote adaptive changes in the nervous system, enhancing neuronal plasticity and synaptic efficiency. This neuroplasticity enhances the brain's fine regulation of motor control, thereby improving flexibility. Situational settings and task-oriented activities in the retraining of children with ASD help improve attention, planning abilities, and working memory. Previous studies have shown that regular participation in physical activities such as swimming and ball sports can significantly enhance coordination and reaction time in children with ASD, thereby boosting cognitive control capabilities.

Exercise interventions not only promote physical health but also positively impact the psychological and social adaptation of children with ASD. Interaction and cooperative behavior during exercise provide valuable social contexts for children with ASD, helping them learn social skills and coping strategies, thereby enhancing cognitive control and flexibility. Coaches can create personalized exercise plans based on individual differences during interventions to ensure that each child trains in a safe and supportive environment. Sensory integration training and physical games can effectively improve the adaptability and independent living skills of children with ASD. The impact of exercise on flexibility and cognitive control in children with ASD is complex and far-reaching. Previous research has indicated that exercise can activate key brain regions such as the prefrontal cortex and hippocampus, which are closely related to cognitive function and emotional regulation. By increasing blood flow and oxygen supply to the brain, exercise promotes the secretion of brain-derived neurotrophic factor (BDNF), enhancing neuronal growth and survival. Additionally, exercise regulates neurotransmitter levels, such as dopamine and serotonin, which play important roles in emotion and cognitive control. Liang (2022) found that regular aerobic exercise significantly improved cognitive flexibility and sustained attention in children with ASD, which may be related to the increase in BDNF levels after exercise. These mechanisms work together to comprehensively enhance the cognitive and behavioral abilities of children with ASD (33).

Through systematic training, it is possible to significantly improve the motor skills and coordination of children with ASD by strengthening specific muscle groups, improving movement patterns, and enhancing neuromuscular coordination. Exercise forms such as balance training, strength training, and aerobic exercise can enhance muscle strength and endurance through repetitive and progressive training, thereby improving children's motor performance. Sensory integration training and occupational therapy within exercise interventions emphasize enhancing the brain's control over the body through multisensory stimulation, improving sensory processing and motor planning abilities in children with ASD. Previous studies have shown that balance board training and core muscle group training can significantly improve both static and dynamic balance in children with ASD, which is crucial for their motor control and coordination in daily activities.

The impact of exercise on motor skills and coordination in children with ASD can be explained by optimizing movement patterns and improving mechanical efficiency (34). Biomechanical research helps design more effective training programs by analyzing joint angles, muscle activity, and mechanical load during movement, maximizing exercise benefits and reducing injury risk (35, 36). Exercise biomechanics further reveal how adjusting posture and movement patterns can enhance movement efficiency (37). For example, running and jumping training can improve gait and landing patterns by enhancing lower limb strength and explosive power, thereby improving motor skills and coordination. Almeida (2021) demonstrated that specific jump training significantly improved vertical jump height and horizontal jump distance in children with ASD, indicating positive effects of exercise training on their explosive power and coordination (38).

The deep mechanisms by which exercise impacts motor skills and coordination in children with ASD are mainly reflected in neural plasticity and physiological adaptation (39). Appropriate training promotes adaptive changes in the central nervous system, enhancing synaptic connectivity efficiency and improving the coordination and integration of neural networks. Optimizing cardiopulmonary function and enhancing blood circulation increase muscle oxygenation and metabolic efficiency, thereby enhancing overall motor performance. Shahane (2024) found in a systematic review that regular aerobic and strength training can improve maximal oxygen uptake and muscle strength in children with ASD, leading to better coordination and motor skills across various tasks (40).

Teamwork and interactive games can significantly enhance the social skills of children with ASD (41). Team sports like soccer and basketball require communication and cooperation among team members, providing opportunities for children with ASD to practice social skills (42). Previous studies have shown that participating in team sports significantly improves cooperation, communication, and conflict resolution in children with ASD (43). Additionally, role-playing and situational simulation activities in exercise training help them understand and adapt to different social contexts, enhancing social adaptability. From a sociological and psychological perspective, exercise not only boosts self-esteem but also reduces social anxiety and promotes emotional expression (44). Exercise activities provide an inclusive environment where children with ASD feel a sense of belonging and recognition, thereby enhancing self-esteem. Psychological research further indicates that exercise can reduce social anxiety and improve emotional stability by releasing endorphins and other neurotransmitters. Activities like aerobic exercise and yoga have been shown to significantly reduce anxiety levels in children with ASD, improving their performance in social situations and promoting the development of emotional expression and empathy, helping them better understand and respond to others' emotions. Furthermore, participating in exercise training enhances physical control and coordination in children with ASD, boosting their confidence and initiative in social interactions, improving response speed and physical flexibility, and helping them perform better in dynamic social situations.

Participating in physical activities can significantly improve social skills and emotional regulation in children with ASD, thereby alleviating behavioral problems. Sociological studies have shown that team sports and interactive games provide rich social contexts, helping these children learn how to interact with others, reducing feelings of isolation and social frustration. Additionally, neurotransmitters like endorphins and dopamine released during exercise can improve emotional stability and reduce symptoms of anxiety and depression. By promoting brain development and enhancing executive functions such as attention, working memory, and cognitive flexibility, exercise can reduce impulsive behaviors and self-injury. From a neurological perspective, exercise enhances the plasticity of the central nervous system, promoting neuron growth and synaptic connectivity, improving brain blood flow and oxygenation levels, thereby enhancing behavioral regulation. Regular aerobic and strength training can significantly increase dopamine and serotonin levels, effectively reducing behavioral problems. These mechanisms work together to comprehensively enhance behavioral performance and quality of life in children with ASD.

The present study still has some research limitations: (1) High Heterogeneity: The high heterogeneity among the included studies may affect the reliability of the results. In this study, the effects differed significantly across different grade levels and intervention forms, leading to overall high heterogeneity. (2) Variation in Intervention Programs: Differences in the forms, frequencies, and durations of intervention programs among the included studies affected the uniformity and comparability of the meta-analysis results. Significant differences in intervention durations and forms in this study impacted the effect evaluation. (3) Sample Size Limitation: Some of the included studies had small sample sizes, reducing the statistical power of the meta-analysis. Insufficient sample sizes may lead to some subgroup analysis results not reaching significance levels. (4) Lack of Long-term Follow-up Data: Most studies lacked long-term follow-up data, making it difficult to evaluate the sustainability of the intervention effects. Although the 8–12 week interventions in this study showed significant effects, the long-term effects were not evaluated.

## Conclusion

This study investigated the effects of exercise interventions on flexibility, cognitive control, motor skills, coordination, social abilities, and behavioral problems in children with ASD. The results showed positive effects of exercise interventions in multiple domains. Although the overall effect on flexibility and cognitive control did not reach statistical significance, upper-grade students showed significant improvement in these areas. The most notable improvements in motor skills and coordination were observed in lower-grade students. Preschool children exhibited significant enhancement in social abilities, and behavioral problems improved significantly across all grade levels. Martial arts and ball sports were particularly effective in these areas.

The findings support exercise interventions as an effective means to improve various abilities in children with ASD and emphasize the importance of designing personalized intervention programs tailored to different ages and needs. The results further confirm the broad positive impact of exercise interventions on children with ASD. Future research should focus on larger sample sizes and long-term follow-ups to further validate the sustainability and generalizability of the intervention effects.
